# Spatial and Temporal Patterns of Ecological Connectivity in the Ethnic Areas, Sichuan Province, China

**DOI:** 10.3390/ijerph191912941

**Published:** 2022-10-10

**Authors:** Shili Guo, Xian Deng, Jiaxuan Ran, Xiangyu Ding

**Affiliations:** 1 School of Economics, Southwestern University of Finance and Economics, Chengdu 611130, China; 2 Office Service Center of Standing Committee of Nei Jiang Municipal People’s Congress, Neijiang 641000, China; 3 China Western Economic Research Center, Southwestern University of Finance and Economics, Chengdu 611130, China

**Keywords:** ecosystem service, ecological connectivity, ecological resistance, minimum cumulative resistance model, spatial and temporal patterns, ethnic areas

## Abstract

With ongoing economic and social development, natural habitats are becoming increasingly fragmented, blocking habitat connections and reducing landscape connectivity. The study of changes in ecological connectivity can provide valuable information for habitat and landscape restoration, which are necessary for sustainable regional development. Despite the growing interest in this issue, studies that reveal the change in ecological connectivity in the compounded areas of ecological vulnerability and deep poverty are still lacking. In this paper, one of the most underdeveloped and ecologically fragile southwestern ethnic regions of China, the Sanzhou region of Sichuan Province, was the study area. Based on a vector map of current land-use status and vector data on ecosystem factors and nature reserves in 2010 and 2015, the change in ecological connectivity was analyzed using the minimum cumulative resistance model using GIS spatial analysis method. Firstly, ecological sources were identified based on the distribution of ecological functional areas. Secondly, the ecological resistance surface based on ecosystem service value is revised by integrating the three dimensions of topography and hydrology, ecological environment and development, and utilization intensity. Finally, the ecological connectivity of ethnic areas in southwest China in 2010 and 2015 was compared and analyzed through the perspective of ecological resistance. The results show that: (1) From 2010 to 2015, the overall ecological connectivity decreased. (2) There were six areas of high ecological resistance featuring human activity and ecological degradation: the Anning River Valley in Liangshan Prefecture, Ganzi, Dege and Luho counties in Ganzi Prefecture, and Ruoergai and Hongyuan counties in Aba Prefecture. (3) Low ecological resistance areas were more numerous and widely distributed, forming an ecological protection barrier for the three autonomous prefectures, and regulating and protecting their natural environments. It is necessary to maintain and strengthen this protection; accordingly, measures are proposed to improve ecological connectivity. This study provides a reference for achieving ecological security and harmonious coexistence between humans and nature in this region.

## 1. Introduction

With the current rapid rates of industrialization and urbanization, humans are degrading the natural environment and exploiting its resources with increasing intensity. Meanwhile, the global Living Planet Index continues to decline [1], with extreme weather and natural disasters intensifying [2]. In this context, China has been paying increasing attention to environmental protection and the harmonious development of humans and nature. In environmental protection, the ecological land landscape type is important for maintaining high-quality ecological environments, biodiversity and dynamically balanced ecosystems [3,4]. In contrast, ecosystems usually involve material cycling, energy flow and information transfer between components in the form of flow patterns. The directions, paths and velocities of these flow patterns have significant impacts on the ecosystem. Ecological flows spread horizontally by aggregation. As they traverse an ecosystem, they exchange and circulate resources within it; however, they need to overcome spatial resistance to do so, and they influence the interactions between their associated resource patches [5,6]. Among them, the extent to which the ecological landscape facilitates or hinders ecological flow is called ecological connectivity [7]. Ecological connectivity is also the basis of ecological processes such as material and energy circulation and exchange, gene interference and dissemination, species migration and dispersal, and soil erosion and infiltration in the regional ecosystem [8,9]. Therefore, ecological connectivity is an indicator of the extent to which the overall landscape hinders (or promotes) the operation of ecological flows. The quality of an ecosystem can be evaluated quantitatively, and the optimization of its patterns contributes to its stability [10,11,12].

In recent years, ecological connectivity has become a focus of research in landscape ecology, conservation biology and other related disciplines [13,14,15,16,17]. Most research has focused on species conservation and landscape patterns. In research on species conservation, the habitats of specific rare species have been defined as the study areas. In the Flexi River Basin of the Sanjiang Plain in Northeast China, Li et al. [18] used the habitat suitability index (HSI) model to analyze the degree of habitat fragmentation and its drivers during the breeding season of the Tandeung crane. In southwest China, giant panda habitats are the main research areas. Chen et al. [19] reported on the fragmentation of giant panda habitat in Wolong Nature Reserve as early as 1999, then Viña et al. [20] and Bu et al. [21] studied the dynamic changes in the ecological connectivity of that habitat in terms of habitat patch identification, wildlife corridors and land cover changes outside the reserve. In landscape pattern research, land-use planning is the main research direction; however, with the increasing global concern for ecological security, landscape ecological security patterns have also become a research hotspot. Among the large number of studies on land use, Li et al. [22] and Darvishi et al. [23] analyzed the impact of land use and cover changes on ecological connectivity from the perspective of land remediation. They also proposed ways to optimize ecological patterns. Optimization of wetland landscape patterns has mainly focused on changes in fish habitats [24], waterfront areas [25] and river mouths [26]. Urban green space construction research has emphasized the planning of urban green space systems [27]. In research on ecological safety, most studies have selected study areas that are single administrative units [28,29,30] or geographical units [31,32] while ignoring the cross-compound areas of both.

Therefore, after reviewing the literature related to ecological connectivity, this study integrated the research perspectives of landscape patterns and species conservation. The study region was a composite administrative and geographic space: the Sanzhou region of Sichuan Province, namely, the Aba Tibetan and Qiang Autonomous Prefecture, Ganzi Tibetan Autonomous Prefecture and Liangshan Yi Autonomous Prefecture. Due to their unique geographical locations and environments, these areas are ecologically fragile and have key ecological functions, including active material cycling, energy flows and species migration. They also contain human residents from ethnic minorities who live in poverty; therefore, the social aspects of this study are significant.

Methods for quantitatively evaluating ecological connectivity mainly comprise connectivity indices and connectivity models. Among them, the more widely used indexes are the integral index of connectivity (IIC) and the probability of connectivity, PC (PC) [33,34]. These two indices not only better reflect the fragmentation of the landscape and identify stepping stone patches, but also consider the dispersal process of organisms to evaluate the impact of each element of the landscape on ecological processes from a functional point of view and to better measure the ecological connectivity [33]. With the ongoing study of ecological connectivity, a variety of models have been applied, such as Dynamic Model of the Landscape, Composite Population Model and Migration Diffusion Model [35]. Among them, Dutch ecologists proposed the minimum cumulative resistance model [35], which became a widely used connectivity model, and it itself can be used as a quantitative indicator of the landscape resistance function. Minor and Urban [36] explored the importance of patch size on ecological connectivity, pointing out that maintaining high connectivity in highly fragmented landscapes benefits wildlife migration and dispersal. Levin et al. [37] used a modified minimum cumulative resistance model to analyze connectivity at the national and regional scales in Israel, affirming the important role of open geographic space in ecological conservation. In China, the minimum cumulative resistance model has also been widely used in ecological connectivity studies. Wang et al. [38] used the minimum depletion distance as a quantitative index of landscape connectivity to analyze the ecological connectivity between landscapes. Li et al. [22] used the minimum cumulative resistance model to analyze the impact of land remediation on ecological connectivity and proposed a way to optimize ecological patterns. Chen et al. [39] used the minimum depletion distance model to calculate the ecological service value of land and an ecological connectivity index, and extensively analyzed the influences of ecological connectivity. Therefore, in quantitative research into ecological connectivity, methods are selected according to the characteristics of the study area and the specific research requirements; nevertheless, the main research method is based on the minimum cumulative resistance model.

This study focuses on the Sanzhou region of Sichuan Province, China, which is a complex of administrative space and geographic boundaries, and is not only similar in geographic location and natural environment but is also an administrative aggregate. The Sanzhou region is a core area used for constructing an ecological security barrier in the upper reaches of the Yellow River. It has important ecological functions, such as water connotation, soil conservation, wind and sand control, and biodiversity maintenance. It is also an area where ethnic minorities reside in contiguous special hardship areas under conditions of deep poverty. Hence, the ecological environment and economic development are inextricably linked, so environmental protection issues in this region have received much ecological, geographic and economic research attention. Accordingly, this study considers the topography, hydrology, ecology, development and utilization intensity characteristics of the study area. In this paper, based on a vector map of the current land-use status and vector data on ecosystem factors and nature reserves in 2010 and 2015, the change in ecological connectivity was analyzed using the minimum cumulative resistance model by using GIS spatial analysis method. Firstly, ecological sources were identified based on the distribution of ecological functional areas. Secondly, the ecological resistance surface based on ecosystem service value is revised by integrating the three dimensions of terrain and hydrology, ecological environment and development and utilization intensity. Finally, the ecological connectivity of ethnic areas in southwest China in 2010 and 2015 was compared and analyzed through the perspective of ecological resistance. This provides the foundations for government’s policy decisions for achieving ecological security and harmonious coexistence between humans and nature in this region.

In this research, we respond to the following questions, i.e., how to: (1) construct a minimum cumulative resistance model and calculate ecological resistance values so as to measure the ecological connectivity in the compounded areas of ecological vulnerability and deep poverty; (2) explain the spatial and temporal variability of ecological connectivity in the study area. Our main contributions are: (1) providing a new research perspective (ecological connectivity) to measure ecosystem quality and integrating multiple data sources into a consistent methodological framework for measuring ecological connectivity; (2) selecting a study area with Chinese characteristics, i.e., the Sanzhou region of Sichuan Province, which has multiple considerations of ecological security, socioeconomic development and ethnic minorities.

This paper is organized as follows. Section 2 illustrates the study area, data sources, and the rationale and parts of the methodological framework, which mainly includes identifying ecological sources and constructing ecological buffer zones (Section 2.2.1), constructing ecological resistance surfaces (Section 2.2.2), classification of Indicators and assignment of Values (Section 2.2.3) and ecological resistance surface calculation (Section 2.2.4). In Section 3, we discuss the results of this study. In Section 4, we suggest corresponding countermeasures and some research shortcomings of our findings, and open up future research directions. In Section 5, we summarize the main messages of our work and present our conclusions.

## 2. Materials and Methods

### 2.1. Study Area and Data Sources

Located in the hinterland of southwest China, Sichuan Province is multi-ethnic, containing the largest Yi settlement and the only Qiang settlement in China, as well as the second-largest area of Tibetan residents. Geographically, Sichuan Province is located within the ecological protective screen of the Qinghai-Tibet Plateau and the Loess Plateau-Chuanian-Tianduan. It is an important water conservation area of the upper reaches of the Yangtze River, and an important water recharge area for the upper reaches of the Yellow River. It is a global biodiversity conservation hotspot and occupies an important position in the national ecological security strategy. The Northwest Sichuan Ecological Demonstration Zone (part of the Sichuan Ecological Environment Zoning Control Program) actively explores the mechanisms and path of the “two mountains” (“Building an ecological civilization is vital to sustain the Chinese nation’s development. We must realize that lucid waters and lush mountains are invaluable assets and act on this understanding”, colloquially known as the “Two Mountains” theory) transformation in order to promote the transformation of ecological product value and, at the same time, the problems of ecosystem functional degradation, the mismatch between the supply and demand of ecosystem services, disorder and imbalance in spatial patterns, and low efficiency of resource utilization. The Northwest Sichuan Ecological Demonstration Area is located in northwestern Sichuan Province, and is adjacent to Qinghai and Gansu Provinces to the north, Tibet Autonomous Region to the west, the Chengdu Plain and Northeast Sichuan Economic Zones to the east, and borders Yunnan Province and the Panxi Economic Zone to the south. The Ganzi Tibetan Autonomous Prefecture and Aba Tibetan and Qiang Autonomous Prefecture contain a total of 31 counties and cover an area of 232,600 km^2^, accounting for 47.8% of Sichuan Province. The region is sparsely populated and has a relatively low level of economic development, with a population density of <10 people/km^2^. In 2018, the region’s residential population of 2.14 million accounted for only 2.6% of the province’s total population. The Tibetan population comprises 78% of the population. The urbanization rate is only 35%, significantly less than the province’s average. The gross regional product is 59.79 billion yuan, accounting for only 1.5% of the province’s total. The GDP per capita is 28,000 yuan, equivalent to 57% of the province’s average.

**Figure 1:**
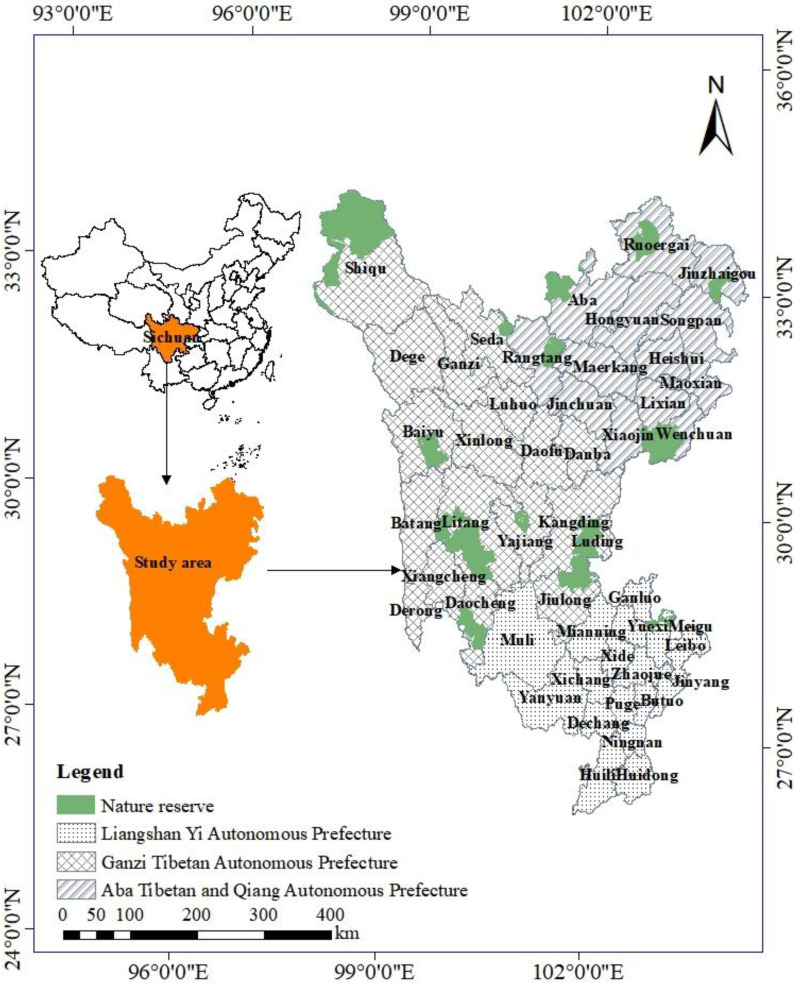
Map of the study area and ecological source sites.

The Sanzhou region of Sichuan Province is located in the western part of Sichuan Province and at the eastern edge of the Qinghai-Tibet Plateau. It includes Ganzi Tibetan Autonomous Prefecture, Aba Tibetan Autonomous Prefecture and Liangshan Yi Autonomous Prefecture and, geographically, greatly overlaps the Northwest Sichuan Ecological Demonstration Area. The Sanzhou region is vast, spanning 26°03′–34°20′ N latitude and 97°22′–103°52′ E longitude, with a total area of 29.73 km^2^, accounting for 61% of the province. It has three county-level cities and 45 counties under its jurisdiction (Figure 1). The geographical location of Sanzhou region is precarious and it is rich in natural resources. Ganzi Prefecture is located in the transition zone from the western edge of the Sichuan Basin to the Tibetan Plateau, spanning 27°58′–34°20′ N latitude and 97°22′–102°29′ E longitude. The average altitude is about 3500 m and has high terrain, rich hydrology, 139.783 billion m3 of water resources and 146,100 km^2^ of river basins. Some 61.7% of its total area is natural grassland and it contains 20% of the total forest area of Sichuan Province. Aba Prefecture is located at the southeastern edge of the Tibetan Plateau spanning 32°18′–33°37′ N latitude and 101°18′–102°35′ E longitude, and has a typical plateau topography. The average altitude is between 3500 and 4000 m and there are many rivers and lakes in the territory, with an average total of 44.6 billion m^3^ of water resources. Liangshan Yi Autonomous Prefecture is located in the southwest of Sichuan Province, spanning 26°03′–29°18′ N latitude and 100°03′–103°52′ E longitude. The average altitude is about 3100 m and the technically exploitable amount of hydro energy resources is more than 70 million kilowatts.

The data used in this study mainly included current land-use data, administrative boundary vector maps, topographic and geomorphological (DEM) images, nature reserve vector maps, and socioeconomic data of the three states in Sichuan Province from 2010 to 2015. Among them, current land-use data of the three states with a spatial resolution of 1 km were obtained from the Resource and Environment Science Data Center of the Chinese Academy of Sciences (http://www.resdc.cn/ accessed on 5 March 2018), which also provided administrative boundary vector maps, DEM data and ecosystem service value data.

### 2.2. Methods

**Figure 2:**
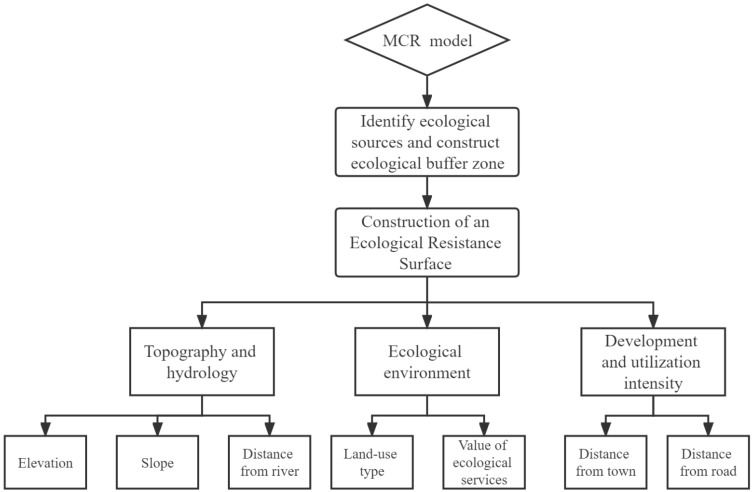
Diagram of the research procedures.

In this paper, a minimum cumulative cost distance (MCR) model was used to analyze ecological connectivity changes, in which source and resistance surface are its two most basic concepts. The source refers to the cost center of a function, while the resistance surface is the landscape resistance that needs to be overcome to achieve a certain ecological process [40]. Based on the results of relevant domestic and foreign studies, and combined with the vector maps of the current land-use situation in 2010 and 2015 in the Sanzhou area and the national-level data on ecosystem service values, spatial analysis methods (such as superposition analysis and buffer zone analysis in GIS) were used to (1) determine ecological sources, (2) construct an index from three dimensions of (i) topography and hydrology, (ii) ecological environment and development and (iii) utilization intensity, and (3) apply the minimum cumulative resistance model. This generated a resistance surface and calculated the ecological resistance values so as to evaluate the ecological connectivity of the three states. The results provide a scientific basis for guaranteeing ecological security and the sustainable and coordinated development of the three states. The main processes are as follows (Figure 2).

#### 2.2.1. Identify Ecological Sources and Construct Ecological Buffer Zones

Ecological “source” sites are the origin of species dispersal and maintenance, and can comprise species-specific habitats or suitable areas [41]. In this paper, 29 nature reserves in three states of Sichuan Province (Figure 1) were selected as ecological source sites. These perform the roles of maintaining the sustainability of ecosystem services, increasing ecological functionality and maintaining the stability of the region’s ecosystems. The source sites were used to build a resistance surface to evaluate the ecological connectivity of the study area.

A buffer zone is a low cumulative resistance zone around ecological sources, which is used to protect the ecological process and natural succession in the core area, and reduce the impact caused by human disturbance of the external landscape [42]. Based on the minimum cumulative resistance surface, the areas outside the ecological source are divided into regions. Loss et al. [43] found that the density of silkworm, songbird and other animals was the highest within 500 m outside a nature reserve in Chicago. Ichinose [44] concluded that bird diversity was related to the proportion of forest area within 500 m around a park in Kobe. Taking this spatial distance as a reference, based on the accumulated consumption surface generated by the ecological source and comprehensive resistance surface, a certain spatial range at the edge of the ecological source is taken as the buffer zone. In view of the different land types around the source, it is necessary to constantly adjust the discontinuity to ensure that the narrowest part of the buffer zone is wider than 500 m.

#### 2.2.2. Construction of an Ecological Resistance Surface

The resistance for ecological flow between different landscape units is mainly affected by landscape types, natural and human disturbances and landscape connectivity. With consideration of the present situation and data availability of the three states of Sichuan Province, three dimensions were used to measure ecological resistance: topography and hydrology, the ecological environment and exploitation intensity [45,46,47,48]. Indicators of resistance factors were selected for each dimension in such a way that each indicator was required to have a minimal correlation with other indicators that represented the significance of their different impacts. The topographic and hydrological dimension was represented by elevation, slope and distance from rivers; the ecological environment dimension was represented by ecological service values and land-use types; and the development and utilization intensity dimension were represented by distance from major roads and distance from towns. For these, weights were obtained by drawing on previous studies and expert scoring [36,49,50,51] (Table 1).

#### 2.2.3. Classification of Indicators and Assignment of Values

##### Topography and Hydrology Dimension

This dimension was evaluated by elevation, slope and distance from rivers. The grade settings and resistance values for each evaluation factor are shown in Table 2.

##### Elevation

Elevation affects the spatial distribution of land resources and the way they are used. Generally speaking, as elevation increases, the living environment becomes worse and the costs of construction and development increase. The overall density of farming settlements will be lower at higher elevations. Hence, higher areas are less affected by humans, which promotes their natural habitats. The higher the elevation, the lower the ecological resistance and the stronger the ecological connectivity [52,53,54,55].

**Figure 3:**
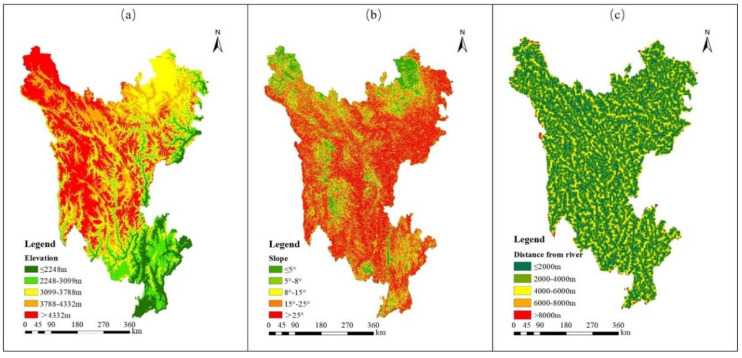
Characteristics of assessment indicators for topography and hydrology dimension in the study area. (**a**) Ranks of the elevation; (**b**) Ranks of the slope; (**c**) Ranks of the distance from river.

As the elevation of the study area ranged from 379 m to 7000 m, the elevation-dependent variation in land use distribution is difficult to evaluate when the elevation gradient is divided in an oversized or equally spaced manner without significant differences [56]. To characterize the influence of elevation on land-use types, the natural breakpoint method in ArcGis 10.2 was used to divide the elevation of the study area into five levels: Level 5 ≤ 2248 m and its resistance value = 5; Level 4 = 2248–3099 m, resistance = 4; Level 3 = 3099–3788 m, resistance = 3; Level 2 = 3788–4332 m, resistance = 2; Level 1 > 4332 m, resistance =1. Among the three states in Sichuan Province, the elevation of Ganzi was mostly > 4332 m, followed by that of Aba, while the elevation of Liangshan was the lowest. The elevation of most areas was <3788 m, as shown in Figure 3a.

##### Slope

The gradient of a slope constrains the distribution of land resources and how they are used. The degree of soil erosion is often related to slope, which can also have important impacts on seed dispersal and energy flow processes. Generally speaking, areas with relatively gentle ground have a better foundation tolerance, often without natural vegetation. Hence, they are more suitable for human development and are more influenced by human factors. On the contrary, areas with relatively steep ground retain less soil and water, are more permeable to soil and are more suitable as forest and pasture areas. Meanwhile, areas with steep ground often have high peaks, diverse vegetation and streams and are more suitable for establishing nature reserves to maintain the species diversity of the area. Thus, slope influences the expansion of ecological land. The greater the slope, the smoother the spatial ecological flow; the lower the ecological resistance, the stronger the ecological connectivity.

Slope data for the three states were extracted from the DEM data using the slope tool in ArcGis 10.2, and ranged from 0° to 42°. The slope resistance factor of the ecological land was divided into grades: slope ≤5° = grade 5, which was assigned a relative resistance value of 5; 5–8° = grade 4, relative resistance = 4; 8–15° = grade 3, relative resistance = 3; 15–25° = grade 2, relative resistance =2; and slope > 25° = grade 1, relative resistance = 1 (Figure 3b).

##### Distance from River

Rivers provide water, which is essential for survival and development. They have the roles of environmental purification, improving ecological functioning and maintaining ecological stability. The water system network is well developed and water resources are abundant in the Sanzhou region of Sichuan Province. There are rivers and lakes in this region, which have positive impacts on ecological land, so a hydrological factor should be considered. Locations closer to a river have richer water resources, more favorable expansion of ecological sources and stronger ecological connectivity. In this paper, hydrological analysis was based on DEM elevation data. The distance of ecological land from rivers was obtained using Euclidean distances.

Based on the distribution of rivers in the Sanzhou region, the distance from rivers was divided into five resistance factors: Level 5 = 8–10 km, resistance value = 5; Level 4 = 6–8 km, resistance = 4; Level 3 = 4–6 km, resistance = 3; Level 2 = 2–4, resistance = 2; and Level 1 ≤ 2 km, resistance = 1 (Figure 3c).

##### Ecological Environment Dimension

The ecological environment dimension is represented by ecological service value and land-use type. The level settings and resistance values for each evaluation factor are shown in Table 3.

##### Land-Use Type

Different human uses of land resources have different degrees of influence on the exchange of materials, energy and information involved in the process of ecological land expansion. Switching land-use types can change surface vegetation, reduce land fertility and degrade ecological functions. Therefore, land-use type is an extremely important resistance factor in the process of ecological source expansion. In this study, current land-use data for the three states were extracted from data on Sichuan Province in 2010 and 2015, downloaded from the Resource and Environment Science Data Center of the Chinese Academy of Sciences. A mask cropping tool was used to create an administrative boundary vector map of the three states.

**Figure 4:**
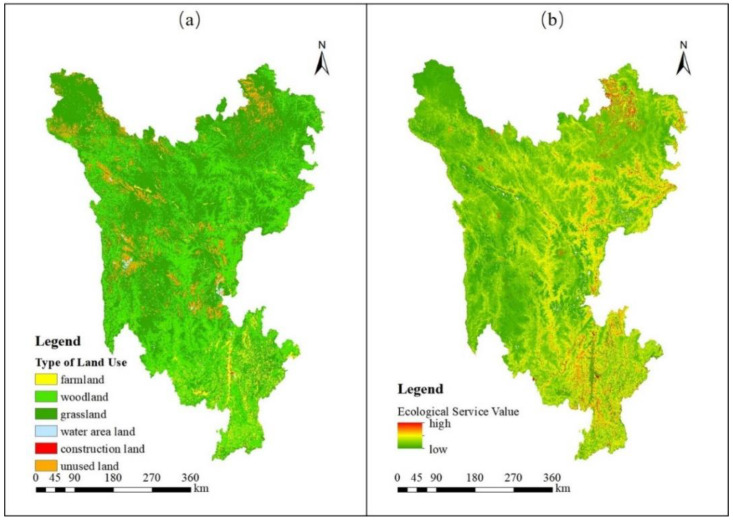
Characteristics of assessment indicators for ecological environment dimension in the study area. (**a**)The type of land use; (**b**) The ecological service value.

A reclassification tool was then used to classify the land-use types into five categories: watersheds, forest land, grassland, cropland, unbuilt land and built land. According to previous studies, each type of land use in the Sanzhou region of Sichuan Province was assigned a resistance coefficient: woodland and watersheds = 1; grassland = 2; cropland and unbuilt land = 3; and built land (which often contains human activities and features the greatest ecological damage and pollution) = 5 (Figure 4a).

##### Value of Ecological Services

Ecosystem service value refers to the benefits that humans derive directly or indirectly from ecosystem functions. The purpose of human activities in ecosystems is to derive benefits, including product supply, aesthetic perception and safe shelter [57]. The value of ecological services quantifies the human-ecosystem interactions and is an important indicator of the stability of ecosystems and the maintenance of ecological security patterns.

Ecological Services (ES) are life support products and services obtained directly or indirectly through the structure, processes and functions of ecosystems. In 1997, on the basis of Costanza’s assessment of global ecological assets [58], Xie et al. [59] developed a table of ecological service value equivalence factors for ecosystems in China, and pointed out that the size of ecological service function of an ecosystem is closely related to the biomass of that ecosystem. This method has been widely adopted and cited.

China’s terrestrial ecosystem service value data is based on the national remote sensing classification of terrestrial ecosystem types, and the ecosystem types include: dryland, farmland, coniferous forest, mixed coniferous forest, broadleaf forest, shrubland, grassland, scrub, meadow, wetland, desert, bare land, water system, glacial snow, artificial surface (including construction land, industrial and mining land), 15 secondary categories (farmland, forest, grassland, wetland, etc.), and 6 primary categories of desert and water. Based on the spatial distribution of NPP, precipitation and soil conservation, the value of each ecosystem service equivalent factor was adjusted by referring to the ecological service equivalent factor method [59], and the values of national food production, raw material production, water supply, gas regulation, climate regulation, environmental purification, hydrological regulation, soil conservation, nutrient cycle maintenance, biodiversity and aesthetic landscape were calculated (Table 4).

The values of 11 ecological services were calculated. In theory, higher ecosystem service values are associated with greater service functions, easier material-energy transport between landscape units and smoother ecological flow. Therefore, based on ecosystem service value data from 2010 to 2015, an ecological service value index was constructed with three levels: high, medium and low, with resistance values of 1, 2 and 3, respectively (Figure 4b).

##### Development and Utilization Intensity Dimension

The development and utilization intensity dimension is represented by the distances from main roads and towns. The levels and resistance values of each evaluation factor are shown in Table 5.

##### Distance from Town

**Figure 5:**
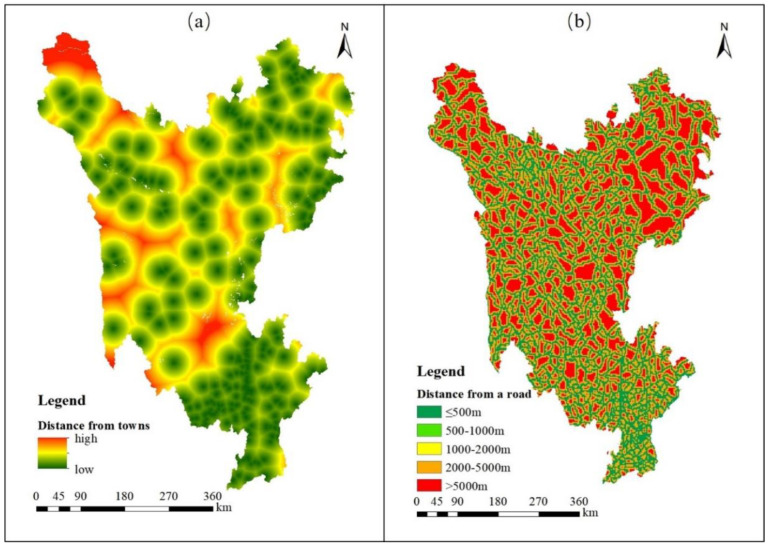
Characteristics of assessment indicators for development and utilization intensity dimension in the study area. (**a**) Ranks of the distance from town; (**b**) Ranks of the distance from road.

Towns and cities are usually the political, economic and social centers of a region. They have a dense population and dense resources and, thus, are more influenced by human activities than other areas. Due to the high population density, human development and utilization of the surrounding land are more frequent, and the surrounding land is more likely to be polluted and damaged. Therefore, the closer an ecological source site is to a town, the less conducive to expansion. In this study, the center of each county was taken as the center of a circle, and the buffer zone analysis function was used to establish buffer zones related to distance from every town within that county. The distances of ecological source sites from the center of each county were divided into five classes [46]. Level 5 represents ≤ 2 km distance; Level 4 = 2–3 km; Level 3 = 3–4 km; Level 2 = 4–5 km; and Level 1 = >5 km (Figure 5a).

##### Distance from Road

Transportation infrastructure stimulates and promotes development of the surrounding land, thus changing the land-use structure and regional landscape pattern. Generally speaking, ecological source sites close to a road are more prone to development than sites further away. In this study, the main traffic arteries, such as national and provincial roads, were identified in the Sanzhou region. Then, the centerlines of the main traffic arteries were used in the Arcgis 10.2 spatial analysis module to establish buffer zones. The distance of an ecological source site from a main road was divided into five levels [60]: Level 5 = ≤0.5 km, resistance value = 5; Level 4 = 0.5–1 km, resistance = 4; Level 3 = 1–2 km, resistance = 3; Level 2 = 2–5 km, resistance = 2; and Level 1 = >5 km, resistance =1 (Figure 5b).

#### 2.2.4. Ecological Resistance Surface Calculation

Ecological connectivity indicates the extent to which ecological land facilitates or hinders ecological processes. The minimum cumulative resistance model was first applied in biomigration studies to describe the processes by which the landscape hinders or facilitates material flows between habitat patches, which is similar to the principle of ecological connectivity [61]. Therefore, ecological connectivity can be converted into the resistance that an ecological flow needs to overcome. The lower the resistance, the stronger the connectivity, and vice versa. The selected vector layers of ecological source sites and raster layers of ecological resistance surfaces were imported into the minimum distance consumed module in ArcGis 10.2 to calculate the minimum cumulative resistance values of all ecological source sites in each raster in the three states. Then, minimum cumulative resistance values were derived to obtain an objective evaluation of the ecological connectivity of the three states. A raster with strong ecological connectivity has a smaller minimum cumulative resistance value, and vice versa.

The calculation steps were: (1) Select the Spatial Analysis module in the Toolbox tool of ArcGis 10.2, then select the Minimum Depletion Distance module, and enter the vector layer of the ecological source site as the element source data in the dialog box of the Minimum Depletion Distance module. Next, input the raster layers of each ecological resistance surface and set the calculation formula according to the weights. Then, the raster data of the ecological resistance surface obtained in the previous step is used to select the path of the output minimum depletion distance raster function. Then, the minimum cumulative resistance surface of ecological source land in the three states was obtained. The minimum cumulative resistance model is expressed as follows [45,47]:

(1) $$MCR=fmin\;(\overset{i=m}{\underset{j=n}{\sum }}H_{ij}\times R_{i})$$

where *MCR* was the minimum cumulative resistance value; *f* indicated the positive correlation between the ecological process and the minimum cumulative resistance; *H_ij_* was the spatial distance from the ecological source patch *j* to the landscape unit *i*; and *Ri* was the resistance coefficient of the landscape unit *i* to the species movement.

## 3. Results

The ecological resistance values of the three states in Sichuan Province in 2010 and 2015 were obtained by following the above steps. In 2010, the lowest resistance value in the minimum cumulative resistance surface of the three states was 0, and the highest was 549,189. The high resistance values were mainly distributed in areas bordering the Anning River Valley in Liangshan Prefecture, Aba Prefecture, Ruoerge County, Aba County and Hongyuan County. In 2015, the lowest resistance value in the minimum cumulative resistance surface of the ecological expansion was 0, and the highest resistance value was 647,473.

### 3.1. Evolution in the Spatial and Temporal Patterns of Ecological Circulation

**Figure 6:**
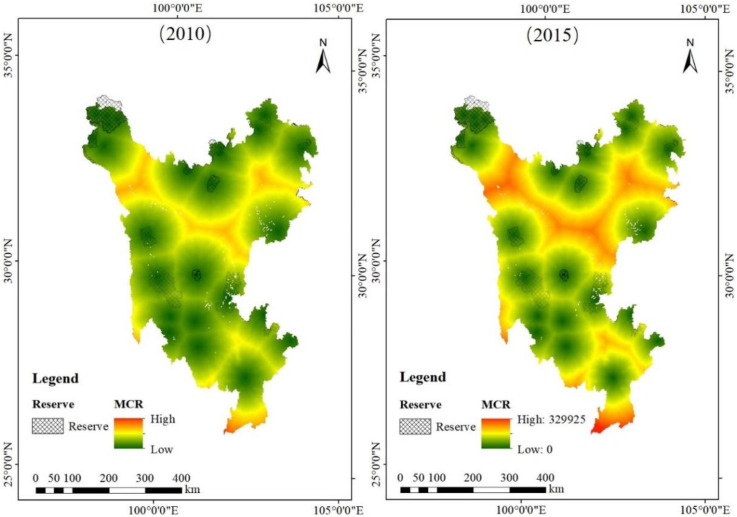
Map of the minimum cumulative resistance surface of the ecological source site.

In 2010, the lowest resistance value in the minimum cumulative resistance surface was 0 and the highest was 549,189 units. In 2010, the area with the highest resistance value in the northern part of the Sanzhou region of Sichuan Province had an inverted “C” circle shape. The resistance values gradually decreased from the middle to the edges in a net shape. The area with high resistance values in the central part was concentrated in the western corner, and the high resistance value distribution was smaller than that in the northern part. The high resistance value area in the southern part was composed of two parts. The central high resistance value area was concentrated in the western corner. Compared with the northern part, the distribution of high resistance values was smaller. The southern area of high resistance values consisted of two parts: part of the inverted “V” circle distribution, with resistance value from south to north gradually decreasing, and the other part located in the bottom of the south, although the distribution range is smaller, the resistance value is the largest resistance value in the Sanzhou area. In 2015, the lowest resistance value in the surface of the minimum cumulative resistance to ecological expansion in the Sanshou region of Sichuan Province remained at 0, and the highest resistance value was 647,473 units, an increase of 98,284 units compared to 2010. Overall, the distribution of resistance values in 2015 in the Sanshou region was largely similar to that in 2010, but the resistance values were higher. This is shown as an increase in the red filled area and a decrease in the yellow and green filled areas (Figure 6).

In terms of geographical location, there were four areas with high resistance values. They were located at the core of the social and economic development area of the Anning River Valley in Liangshan Prefecture, the counties of Ganzi, Dege and Furho in Ganzi Prefecture and in Ruoerge and Hongyuan counties, where the Ruoerge Grassland is located, in Aba Prefecture.

### 3.2. Spatial Clustering of Minimum Cumulative Resistance

**Figure 7:**
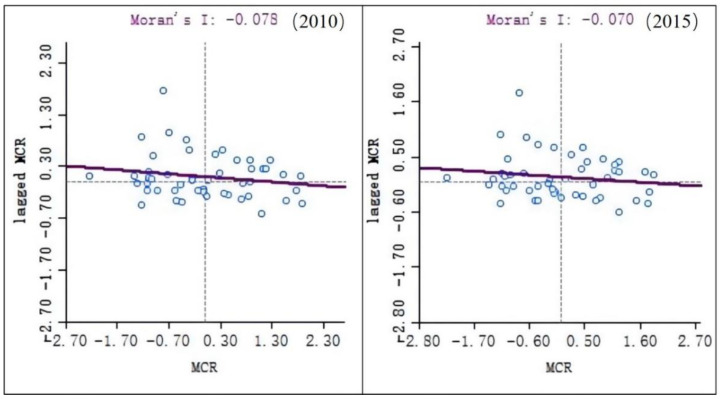
Scattered distribution of minimum cumulative resistance Moran.

Based on the raster data of the minimum cumulative resistance in the three states of Sichuan Province, the average minimum cumulative resistances in each county of the three states were derived using ArcGIS software. Spatial autocorrelation analysis was performed based on these average values. This can visually show the specific distribution of the minimum cumulative resistance among the counties. Figure 7 shows Moran scatterplots of the minimum cumulative resistance in each county in 2010 and 2015. They show that the local Moran indexes were −0.078 in 2010 and −0.070 in 2015. Both of these values were negative, which indicates that the minimum cumulative resistance in each county showed a negative spatial correlation to some extent.

**Figure 8:**
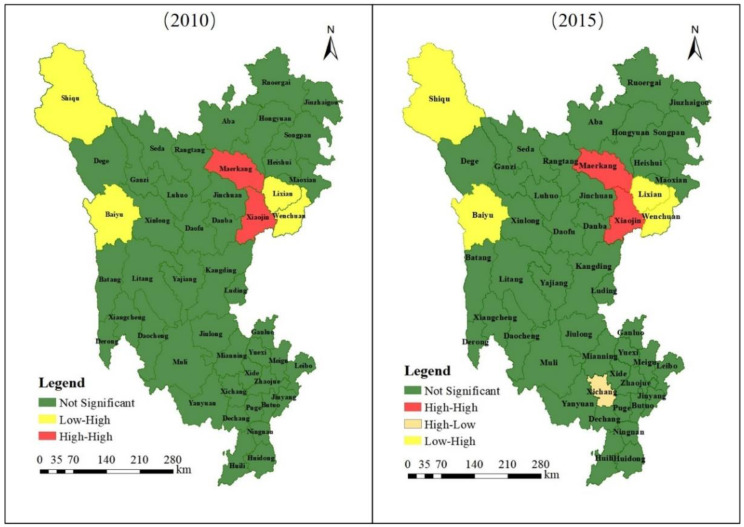
Minimum cumulative resistance LISA aggregation.

The LISA aggregation of the counties in the three states was analyzed using a combination of Geoda and ArcGIS software. The results are shown in Figure 8. High-high aggregation of the minimum cumulative resistance in 2010 mainly occurred in the southern part of Aba Prefecture, including Markang and Xiaojin counties. Low-high aggregation mainly occurred in the southeastern part of Aba Prefecture, including Li and Wenchuan counties, and in the western and northwestern part of Ganzi Prefecture, mainly in Shiqu and Baiyu counties. In 2015, high-high aggregation mainly occurred in the southern part of Aba Prefecture, including Markang and Xiaojin counties, which is consistent with the distribution in 2010. Low-high clustering mainly occurred in the southeastern part of Aba Prefecture, including Li and Wenchuan counties, and the western and northwestern parts of Ganzi Prefecture, mainly in Shiqu and Baiyu counties, which is consistent with the distribution in 2010. High-low clustering occurred in the central part of Liangshan Prefecture in Xichang City. In general, from 2010 to 2015, the minimum cumulative resistance in high-high aggregation area and low-high aggregation area in each county of the three states of Sichuan Province remained basically unchanged, with a high-low aggregation pattern occurring in Xichang City of Liangshan Prefecture in 2015.

## 4. Discussion

In this section, we discuss the results concerning the research questions raised in the introduction. As for the first research question (construct a minimum cumulative resistance model and calculate ecological resistance values so as to measure the ecological connectivity), firstly, ecological sources and zones were identified based on the distribution of ecological functional areas, which is consistent with Xu et al. [62] and Yan et al. [63]. In particular, the Ruoerge Wetland National Nature Reserve and Wolong National Nature Reserve in the Sanzhou region of Sichuan Province have been hotspots of research in the field of ecology [64,65,66,67]. In addition, studies on habitat quality changes also involve the Three Gorges Reservoir Area, the National Ecological Protection Pilot Area and planted forest vegetation [68,69,70]. Secondly, the ecological resistance surface based on ecosystem service value is revised by integrating the three dimensions of topography and hydrology, ecological environment and development and utilization intensity. In this respect, the construction of ecological resistance surfaces remains an open question, as there is no agreement on a clear methodological framework [71,72]. From the existing studies, most of the ecological resistance surfaces are constructed based on the value of ecosystem services. In this paper, the ecological resistance surfaces are modified based on the actual situation in the study area, integrating natural and human factors, with specific indicators including elevation, slope, distance from rivers, ecological service value, distance from towns and distance from roads. Finally, ecological resistance values for the study area in 2010 and 2015 were calculated using GIS spatial analysis methods. Thus, our results provide a more comprehensive and objective picture for analysts and provide a Chinese case study for the development of ecological landscape patterns in the world.

The second research question was to explain the spatial and temporal variation of ecological connectivity in the study area. We calculated the ecological resistance values in 2010 and 2015 in the three states of Sichuan Province through a minimum cumulative resistance model to compare and analyze the spatial and temporal variation of ecological connectivity. From the time dimension, in 2010, the lowest resistance value in the minimum cumulative resistance surface was 0 and the highest was 549,189 units. In 2015, the lowest resistance value in the surface of the minimum cumulative resistance to ecological expansion in the Sanshou region of Sichuan Province remained at 0, and the highest resistance value was 647,473 units, an increase of 98,284 units compared to 2010. From the spatial dimension, the high resistance value areas are mainly distributed in the core of the social and economic development area of the Anning River Basin in Liangshan Prefecture, Ganzi County, Dege County and Furho County in Ganzi Prefecture and Ruoerge County and Hongyuan County, where the Ruoerge Grassland is located, in Aba Prefecture. While the low resistance value areas are widely distributed, most of these areas have nature reserves distributed in between, indicating that setting up nature reserves can effectively restore the ecosystem. From the high resistance value concentration area, the Anning River Valley in Liangshan Prefecture, with relatively good socioeconomic base, location conditions and transportation accessibility [73], has the main concentration of population in Liangshan Prefecture, and, thus, is more influenced by human activities. From 1994 to 2009, the sandy area in Ruoerge County expanded at a rate of 11.08% per year. The sandy area of grassland and wetland increased, and its plant community and soil nutrients had significant decreasing trends [74,75] caused by temperature increases, while human interference was exacerbated by rapid population growth. The government needs to actively implement policies such as returning farmland to forests, returning grazing (farming) land to wetland and conserving biodiversity. These have contributed to increases in wetland and grassland areas, hindering its resistance value to be on a decreasing trend and increasing ecological connectivity. Currently, REDD+ activities advocate the reduction of greenhouse gas emissions through the strengthening of protected areas, which is consistent with the strengthening of ecological connectivity [2].

In view of the evolution in the spatial and temporal patterns of ecological connectivity in the three states of Sichuan Province in 2010 and 2015, this paper makes the following two policy recommendations: (1) Strengthening ecological protection and ecological connectivity. The combination of climate change and anthropogenic activity has led to the ecological degradation of wetlands and desertification of the Ruoerge grassland. Ecological degradation decreases the quality of the natural environment, which is an important cause of the weak ecological connectivity and high ecological resistance observed in the county where the Ruoergei grassland is located. Therefore, on the one hand, we must promote the sustainability of natural resources by establishing nature reserves and wetland parks to stop the reduction of grassland and wetland areas, and strictly prohibit the unreasonable over-exploitation of natural resources. On the other hand, we must establish effective ecological protection mechanisms that are compatible with the legal system. The government must also introduce regulations to provide the necessary legal support for ecological protection. (2) Adjusting the structure of agriculture to reduce interference with the natural environment. It is clear that one of the causes of the high ecological resistance observed in the present study is interference from human activity. Therefore, it is necessary to adjust the structure of agriculture by developing ecological and green agricultural techniques that minimize the adverse effects of farming on the natural environment, thus improving ecological quality and promoting ecosystem sustainability. Taking grassland restoration as an example, Sichuan Province will establish a unified basic grassland management database for the whole province, grasp the management of grazing bans and livestock balance, explore the establishment of a number of grassland natural parks, prepare and implement a pilot program for grassland ecological restoration in northwest Sichuan and take comprehensive measures, such as artificial grass planting, grazing bans, rodent and insect management, to promote grassland ecological management. At the same time, it will establish modern grass production bases and modern grass parks that are integrated with grass and livestock, vigorously promote innovation in the grass seed industry, strengthen the selection and breeding of native grass species and actively explore the development of edible grass.

In this paper, due to data availability, the evaluation indexes of ecological connectivity were only considered from three aspects: topography and hydrology, the ecological environment and exploitation intensity characteristics. Resistance factors such as soil erosion and vegetation cover indexes were not considered, but should be in future research to facilitate the wider application of such evaluation techniques. In addition, this study used Landsat remote sensing data to obtain a land-use classification map of three states in Sichuan Province. Due to the limited accuracy of the supervised classification of remote sensing data, there were some differences between the classification images and the ground truth. In addition, there were some subjective aspects in determining the selection factors and assigning weights to the resistance surfaces in the MCR model, despite the use of an expert scoring mechanism. In future, it is possible to focus on more microscopic study areas, such as the Three Gorges Reservoir Area, the National Ecological Protection Pilot Area and planted forest vegetation [68,69,70]. Changes in ecosystem quality, outcomes and efficiency are understood through more specific and nuanced studies.

## 5. Conclusions

In this study, we selected the Sanzhou region of Sichuan Province as the study area, and analyzed the changes in ecological connectivity using a minimum cumulative resistance model. We also proposed specific restoration and optimization suggestions from two dimensions: strengthening ecological protection and ecological connectivity, and adjusting the structure of agriculture to reduce interference with the natural environment. The results show that:

(1) From 2010 to 2015, the overall ecological connectivity decreased. This is mainly reflected in the larger area and wider distribution of the high-resistance areas, while the increased resistance of the low-resistance areas leads to a more fragmented distribution. The spatial distribution of connectivity is clearly different because the resistance required to overcome the flow of ecosystem material, energy and biological information in landscapes with different ecological services is significantly lower than in human-made landscapes.
(2) There are six areas of high ecological resistance characterized by human activities and ecological degradation: the Anning River Valley in Liangshan Prefecture, Ganzi, Dege and Luding counties in Ganzi Prefecture and Ruoerge and Hongyuan counties in Aba Prefecture. Low-value connectivity areas are concentrated in areas with strong anthropogenic disturbances, such as urbanization, rural settlements, industrial and mining activities, transportation and construction.
(3) Low-resistance areas are more numerous and widely distributed, forming an ecological protection barrier for the three autonomous prefectures, regulating and protecting their natural environment. The high-value connectivity areas are concentrated in high-altitude plateau areas, where there is little anthropogenic disturbance and high ecological service value. Most of these areas have nature reserves distributed among them, indicating that the installation of nature reserves can effectively restore the ecosystem.

## Figures and Tables

**Table 1:** Ecological resistance surface index parameters.

Resistance Factor	Evaluation Factor	Weight
Topography and hydrology	Elevation	0.04
	Slope	0.04
	Distance from river	0.12
Ecological environment	Land-use type	0.14
	Value of ecological services	0.16
Development and utilization intensity	Distance from town	0.28
	Distance from road	0.12

**Table 2:** Ranks and resistance values of each evaluation factor of the topographic and hydrological dimensions of the study area.

Resistance Factor	Evaluation Factor	Level	Resistance Value
Topography and hydrology	Elevation	≤2248 m	5
		2248–3099 m	4
		3099–3788 m	3
		3788–4332 m	2
		>4332 m	1
	Slope	≤5°	5
		5–8°	4
		8–15°	3
		15–25°	2
		>25°	1
	Distance from river	≤2 km	1
		2–4 km	2
		4–6 km	3
		6–8 km	4
		>8 km	5

**Table 3:** Rank and resistance values of each evaluation factor in the ecological environment dimension of the study area.

Resistance Factor	Evaluation Factor	Level	Resistance Value
Ecological environment	Land-use type	Built Land	5
		Unbuilt Land	3
		Cropland	3
		Grassland	2
		Forest land	1
		Watersheds	1
	Value of ecological services	High	1
		Medium	2
		Low	3

**Table 4:** Classification of ecological service types.

Tier 1 Type	Tier 2 Type	Comparison with Constaza Classification	Definition of Ecological Services
Supply Service	Food production	Food production	Conversion of solar energy into plant and animal products that can be consumed
	Raw material production	Raw material production	Conversion of solar energy into bioenergy for human use in buildings and other applications
Regulation Service	Gas regulation	Gas regulation	Ecosystems maintain the balance of chemical components of the atmosphere, absorbing SO_2_, fluoride, and nitrogen oxides
	Climate regulation	Climate regulation	Regulation of regional climate, such as increasing precipitation and decreasing temperature
	Hydrological regulation	Water conditioning, water supply	Freshwater filtration, retention and storage functions of ecosystems and supply of freshwater
	Waste treatment	Waste disposal	Preparation and biological role in the removal and decomposition of excess nutrients and compounds, dust retention
Support Services	Soil maintenance	Erosion control can maintain sediment, soil formation, nutrient cycling	Organic matter accumulation and the role of vegetative root material and organisms in soil conservation, nutrient cycling and accumulation
	Maintaining biodiversity	Pollination, biological control, habitat, genetic resources	Genetic origin and evolution of wild plants and animals, wild plant and animal habitats
Cultural Services	Providing aesthetic landscapes	Recreation, culture	Landscapes with (potential) recreational use, cultural and artistic value

**Table 5:** Ratings and resistance values of each evaluation factor of development and utilization intensity in the study area.

Resistance Factor	Evaluation Factor	Level	Resistance Value
Development and utilization intensity	Distance from town	≤2 km	5
		2–3 km	4
		3–4 km	3
		4–5 km	2
		>5 km	1
	Distance from road	≤0.5 km	5
		0.5–1 km	4
		1–2 km	3
		2–5 km	2
		>5 km	1

## Data Availability

The data used in this study mainly included current land-use data, administrative boundary vector maps, topographic and geomorphological (DEM) images, nature re-serve vector maps, and socioeconomic data of the three states in Sichuan Province from 2010–2015. Among them, current land-use data of the three states with a spatial resolution of 1 km were obtained from the Resource and Environment Science Data Center of the Chinese Academy of Sciences (http://www.resdc.cn/ accessed on 5 March 2018), which also provided administrative boundary vector maps, DEM data, and ecosystem service value data.
